# Localized prediction of tissue outcome in acute ischemic stroke patients using diffusion- and perfusion-weighted MRI datasets

**DOI:** 10.1371/journal.pone.0241917

**Published:** 2020-11-05

**Authors:** Malte Grosser, Susanne Gellißen, Patrick Borchert, Jan Sedlacik, Jawed Nawabi, Jens Fiehler, Nils D. Forkert

**Affiliations:** 1 Department of Diagnostic and Interventional Neuroradiology, University Medical Center Hamburg-Eppendorf, Hamburg, Germany; 2 Department of Radiology, University of Calgary, Calgary, Canada; Henry Ford Health System, UNITED STATES

## Abstract

**Background:**

An accurate prediction of tissue outcome in acute ischemic stroke patients is of high interest for treatment decision making. To date, various machine learning models have been proposed that combine multi-parametric imaging data for this purpose. However, most of these machine learning models were trained using voxel information extracted from the whole brain, without taking differences in susceptibility to ischemia into account that exist between brain regions. The aim of this study was to develop and evaluate a local tissue outcome prediction approach, which makes predictions using locally trained machine learning models and thus accounts for regional differences.

**Material and methods:**

Multi-parametric MRI data from 99 acute ischemic stroke patients were used for the development and evaluation of the local tissue outcome prediction approach. Diffusion (ADC) and perfusion parameter maps (CBF, CBV, MTT, Tmax) and corresponding follow-up lesion masks for each patient were registered to the MNI brain atlas. Logistic regression (LR) and random forest (RF) models were trained employing a local approach, which makes predictions using models individually trained for each specific voxel position using the corresponding local data. A global approach, which uses a single model trained using all voxels of the brain, was used for comparison. Tissue outcome predictions resulting from the global and local RF and LR models, as well as a combined (hybrid) approach were quantitatively evaluated and compared using the area under the receiver operating characteristic curve (ROC AUC), the Dice coefficient, and the sensitivity and specificity metrics.

**Results:**

Statistical analysis revealed the highest ROC AUC and Dice values for the hybrid approach. With 0.872 (ROC AUC; LR) and 0.353 (Dice; RF), these values were significantly higher (p < 0.01) than the values of the two other approaches. In addition, the local approach achieved the highest sensitivity of 0.448 (LR). Overall, the hybrid approach was only outperformed in sensitivity (LR) by the local approach and in specificity by both other approaches. However, in these cases the effect sizes were comparatively small.

**Conclusion:**

The results of this study suggest that using locally trained machine learning models can lead to better lesion outcome prediction results compared to a single global machine learning model trained using all voxel information independent of the location in the brain.

## Introduction

The acute ischemic stroke is one of the most common causes of death and disability worldwide [1]. It is typically caused by a clot interrupting the blood flow to the brain. The undersupplied brain cells down-regulate their metabolism to prolong survival. Severely affected brain cells represent the ischemic core, which is assumed to be not salvageable. This ischemic core dynamically expands into the undersupplied but not yet severely affected brain regions, the so-called tissue-at-risk [2]. Ultimately, recanalization of the occluded arteries can restore blood flow and rescue brain cells within the tissue-at-risk from later cell death. Thus, early treatment with thrombolysis or thrombectomy for removal of the occlusion is of utmost importance. However, in case of treatment in a later time window, the amount of tissue-at-risk that can be potentially saved may not suffice to justify the contraindications such as an increased hemorrhage risk or usage of limited resources [3–6].

Therefore, an adequate estimation of the disease progress is of high clinical interest. Imaging techniques such as computed tomography perfusion and magnetic resonance imaging (MRI), including diffusion-weighted (DWI) and perfusion-weighted MRI (PWI), are commonly used for clinical decision making. These imaging techniques allow estimating the ischemic core and hypoperfused brain tissue using simple thresholds, whereas the mismatch between the two areas represents the tissue-at-risk. However, due to the high complexity and heterogeneity of stroke, such a threshold-based differentiation is often too inaccurate to make reliable therapy decisions [7–10].

In order to make better use of the information included in the imaging data available, the applicability of various machine learning methods has been in the focus of research in recent years [11]. Machine learning or statistical models, including logistic regression (LR), random forest (RF), and artificial neural networks, have been used in the past to learn relationships between the imaging data acquired at the acute stage and the tissue outcome in follow-up imaging [4, 12, 13]. Most of these previously described methods generate a machine learning or statistical model based on globally extracted voxel-wise information from a retrospectively analyzed database of stroke patients with known stroke lesion outcome. They mainly differ with respect to the configuration of imaging features used for training and testing but also regarding the machine learning model used. However, a global modelling approach essentially assumes that differences in the locations of the affected tissue do not play a relevant role for the tissue outcome. Even though it is well known, that different brain tissue types (white and gray matter) and even different brain regions have different compensation potential and thresholds to endure hypoperfusion [14, 15], to our knowledge, no prediction model that really accounts for this aspect explicitly has been described and evaluated so far.

Therefore, the aim of this work was to develop and evaluate a lesion outcome prediction approach that trains and applies voxel-wise machine learning models for prediction of stroke tissue outcome to account for local variabilities. We hypothesized that such an approach enables lesion outcome predictions with higher accuracy compared to using one simple global modelling approach.

## Materials and methods

### Patients

For the development and evaluation of the local tissue outcome prediction approach, multi-center data of patients with anterior circulation strokes collected from 2006 to 2009 was retrospectively analyzed. The study was approved by the local ethics committees and institutional review boards (University Centre Hamburg-Eppendorf, Germany). For each patient, clinical data such as age, sex, National Institutes of Health Stroke Scale (NIHSS) score at baseline, and stroke side were available. Only patients who had a first-time unilateral stroke and an admission NIHSS greater than 4 were included. All patients received conservative or intravenous thrombolytic treatment.

### Imaging

DWI and PWI images were acquired within 12 hours of stroke symptom onset and a follow-up fluid-attenuated inversion recovery (FLAIR) sequence was acquired between 1 and 7 days after symptom onset. Varying MRI sequence parameters were used at the contributing centers. DWI was acquired using magnetic field gradient strengths of b = 1000s/mm^2^, averaged for 3 to 12 directions, and b = 0 s/mm^2^. The in-slice resolution varied from 0.9×0.9 mm^2^ to 2.0×2.0 mm^2^, while the slice thickness was in the range of 6 to 7 mm. Likewise, the in-slice spatial resolution of the PWI datasets varied from 0.9×0.9 mm^2^ to 2.0×2.0 mm^2^, while the slice thickness was in the range of 6 to 6.5 mm. Finally, the follow-up FLAIR datasets were acquired with an in-slice spatial resolution of 0.45×0.45 mm^2^ to 1.0×1.0 mm^2^ and a slice thickness ranging from 6 mm to 7 mm.

### Image processing

DWI, PWI, and FLAIR sequences were further processed employing the software tool AnToNIa [16], briefly described in the following. First, apparent diffusion coefficient (ADC) maps were calculated from the DWI sequence and used to segment the brain tissue and cerebrospinal fluid (CSF). To separate the ipsi- and contralateral hemispheres, we divided the brain segmentation along a 2D hyperplane, approximating the hemispheric fissure (constructed via two manually defined lines). The PWI sequences were corrected for in-slice motion, before a slice-time correction and temporal interpolation (1 second per frame) were applied. After this, the signal curves were converted to concentration time curves. Arterial input functions were automatically extracted using an atlas-based approach and employed to calculate the following perfusion parameter maps using a block-circulant singular value deconvolution approach (applying a threshold of 0.15): CBF (cerebral blood flow), CBV (cerebral blood volume), MTT (mean transit time), and Tmax (time to maximum of the residue function). Finally, the infarct lesions were segmented in the follow-up FLAIR datasets by two neuroradiologists in consensus.

This procedure resulted in ADC, CBF, CBV, MTT, and Tmax maps, the CSF and brain segmentations, and the segmented follow-up lesions. All of these images were rigidly registered and transformed to the baseline DWI (B0) image. Next, the perfusion parameters were normalized using the corresponding average values determined from the contralateral hemisphere (excluding the CSF segmentation). Subtraction was applied for MTT and Tmax and division for CBF and CBV. In order to develop the local prediction models, all parameter maps were registered to the MNI brain atlas in a final pre-processing step using a non-linear transformation determined by the software package ANTs [17]. The resulting transformation was used to transform the parameter maps, the CSF segmentation, and the follow-up lesion segmentations to the MNI atlas.

All intermediate results were visually checked after each step. Datasets were excluded from further analysis in case of missing clinical data, missing imaging data required for this analysis, low image quality, no or poor contrast agent visibility in PWI sequence, artefacts in the image data, no visible follow-up lesions, or insufficient post-processing results.

### Tissue outcome prediction

The basic idea of the local tissue outcome prediction approach, developed in this work, is to train one machine learning prediction model for each brain tissue (or voxel) coordinate in the MNI atlas reference space. Each of these models is trained exclusively using data from the corresponding voxel position and its close vicinity. However, since not enough lesion data is available to train a robust model at all positions in the MNI atlas space, the approach was extended to also enable predictions for voxel coordinates with insufficient lesion information using a global model.

For each voxel, local training sets were composed using three steps. First, the voxel information for the specific voxel of interest was added directly to the training set if it was not segmented as CSF. Due to the exclusion of CSF voxels and because considerably more patients exhibit no lesion in a voxel in MNI space compared to patients exhibiting a lesion, training local models would only be possible for a small number of voxels in MNI space using the training sets resulting from this first step. Based on the assumption of hemispheric symmetry of the brain, voxel information for the voxel of interest mirrored to the contralateral hemisphere was added in a second step to the training set if it was not segmented as CSF. This was implemented via a shift of the x-axis to the middle of the MNI space, so that the hyperplane along x = 0 corresponds to the hemispheric fissure and hemispheres can be differentiated by the sign of the x-coordinate. Nevertheless, even including information from the mirrored hemisphere does not always lead to training sets with a suitable number of observations overall and number of observations with a lesion outcome to train a local model. In other words, these local training sets are typically highly imbalanced with the number of non-lesion voxels greatly outnumbering the number of lesion voxels. For this reason, the training set for each voxel position was practically composed by identifying the closest lesion and non-lesion voxel for each patient within a sphere around the voxel position of interest, which also includes the mirrored datasets. The aim of this procedure was essentially to add two observations for each patient (one with and one without lesion outcome) to the local training set. However, as the aim of this work was to develop inherently local tissue outcome prediction models, a relatively small radius of √5 mm was empirically selected in this work for the search radius around each voxel position. Practically, this was implemented based on the Euclidean distance: For a voxel at position *x*_0_, *y*_0_, *z*_0_, the distance to another voxel at position *x*_1_, *y*_1_, *z*_1_ was calculated as $d=\sqrt{(}{\left(x_{0}-x_{1}\right)}^{2}+{\left(y_{0}-y_{1}\right)}^{2}+{\left(z_{0}-z_{1}\right)}^{2})$. As a result of this, there are still many voxel positions in MNI atlas space, which do not have a sufficient number of training samples in the lesion group so that no machine learning model can be trained. To overcome this issue, the local model is replaced by a global model at those positions. This global model was trained on data from all voxel positions as usually done in previous research. In this work, a group size threshold of 3 was used for selection of the local vs. the global model at each voxel position. This means that if one of the groups used for training (lesion or non-lesion) contains less than three observations, the global model is used while the local model is used otherwise.

Finally, a hybrid tissue outcome prediction approach was implemented. Therefore, the average of the global and the local prediction for each voxel was calculated. For voxel positions, where the local model was not available, the prediction from the global model was used.

In this work, two different algorithms, namely logistic regression (LR) and random forest (RF), were used for the generation of the local as well as the global prediction models using the normalized imaging perfusion parameters (CBF, CBV, MTT, Tmax), ADC, and available lesion outcome information. The LR model was used due to its simplicity, computational efficiency, and its use in multiple previous studies [18, 19]. The RF model was used as it proved superior to the LR model in recent studies while still being simple and efficient enough to apply in this setup [4]. However, in contrast to LR, the calculation time for large numbers of training voxels rises quite fast for the global RF model. Therefore, the training dataset for the global model was down sampled to 500,000 voxels. For this sampling approach, the training dataset was randomly reduced while keeping the same ratio of lesion and non-lesion voxels as contained in the overall training patient’s data. All tissue outcome predictions were generated in R (version 3.4.2) [20].

### Evaluation

Leave-one-patient-out cross-validation was performed for the evaluation of the three modelling approaches (local, global, and hybrid). This means that for every patient’s predicted tissue-outcome, the underlying models were trained on the voxel-information from all other patients.

The three prediction models were evaluated using the area under the receiver operating characteristic curve (ROC AUC), the Dice similarity metric, the sensitivity, and the specificity, by comparison of the predicted lesion to the real lesion outcome [21, 22]. The Dice coefficient is defined by:

$$D(O_{1},O_{2})=\frac{2∙|P\cap L|}{\left|P|+|L\right|}$$

Whereas *P* denotes the binarised prediction lesion and *L* the registered follow-up lesion segmentation. The Dice can take values between 0 and 1, whereas the value 0 indicates that the binary infarct prediction does not overlap with the actual infarct and a Dice value of 1 reflects a perfect match with the actual infarct. To create binary lesion prediction masks based on the predicted lesion probabilities required for calculation of the Dice coefficients, it is necessary to define an appropriate threshold above which the voxel outcome is to be predicted as lesion and vice versa. Therefore, during each iteration of the leave-one-out cross validation, the threshold that results in the optimal Dice coefficient was calculated on the overall available training data (in steps of 0.01) and applied afterwards to calculate the Dice coefficient on the test set predictions. Since we are interested in a single binary prediction and the optimal thresholds for sensitivity (0) and specificity (1) are opposite to each other, they were calculated using the same threshold that resulted in the optimal Dice coefficient.

The ROC AUC reflects the suitability of the model to discriminate between the two outcome classes independent of a specific threshold. It can be interpreted as the probability that a randomly chosen infarct voxel receives a higher risk than a randomly chosen non-infarct voxel. Hence, a ROC AUC of 1.0 would describe a perfect model, while a ROC AUC of 0.5 would indicate that the model is not advantageous to random guessing.

The Dice and ROC AUC values for each prediction model were statistically evaluated using one-sided pair-wise t-tests. A p-value smaller than 0.05 was considered to indicate a significant difference.

In addition, the mean and median values of the local LR model coefficients were calculated for each MNI structural atlas brain region to obtain indications of variation in the different brain regions (see S1 Table).

## Results

Overall, datasets of 99 patients were included for the final analysis in this study. Due to the required minimum number of lesion and non-lesion voxels in the training data (at least three in each group), local models could be trained and applied at 72.33% of all brain tissue voxel positions in MNI space. The local models benefited in particular from the enrichment design choices for the training data. Without them, local models could only be trained for 32.27% of the brain tissue voxels in MNI space. Adding voxels from the opposite hemisphere alone (*i*.*e*. without adding the search radius) resulted in 46.65% of MNI brain tissue voxel positions for which local models could be trained. By adding the small search radius for training data enrichment alone (*i*.*e*. without adding voxels from the opposite hemisphere), local models could be trained for 59.58% of the MNI brain tissue voxels. In the local approach, predictions at voxel positions with insufficient data to train a local model were substituted by the global model.

### Lesion outcome prediction results

Table 1 shows the quantitative results regarding the ROC AUC values, the Dice coefficients, and the sensitivity and specificity metrics for the three modeling approaches (global, local, hybrid) and two machine learning methods (LR, RF). Regardless of the machine learning method (LR or RF), the hybrid tissue outcome prediction approach performed best in terms of the ROC AUC and the Dice coefficient compared to the local and global approaches. At the same time, the local modeling approach performed better compared to the global modeling approach regarding the ROC AUC for both machine learning methods and better regarding the Dice similarity metric for the LR model while the Dice similarity metric was slightly better for the global RF model compared to the local RF model. Both, the local and the hybrid approach performed better than the global approach regarding the sensitivity, with the LR models performing better than their corresponding RF model counterparts. Only for the specificity of the global approach, higher values were observed compared to the corresponding (LR or RF) local and hybrid approaches. However, the differences of the effect sizes for the latter were relatively small compared to the differences in the effect sizes of the other metrics. Sensitivity and specificity should always be compared together due to their relation to each other. Doing so, the local and hybrid approaches led to better results than the global approach. The highest average ROC AUC value was obtained by the hybrid LR (0.872±0.092) and the highest average Dice coefficient was obtained by the hybrid RF (0.353±0.220). The highest average of ROC AUC values and Dice coefficients was obtained by the hybrid LR model (0.872±0.092; 0.348±0.221) followed by the hybrid RF model (0.859±0.089; 0.353±0.220). These models revealed highly significant improvements (p < 0.01) compared to any global or local model regarding the respective metric with effect sizes of 0.011 (compared to the best local model; ROC AUC), 0.061 (global; ROC AUC), 0.016 (local; Dice), and 0.025 (global; Dice). The highest average of ROC AUC values, Dice coefficients, sensitivity, and specificity was obtained by the hybrid LR (0.872±0.092; 0.348±0.221; 0.444±0.252; 0.955±0.047) followed by the local LR, the hybrid and local RF, and the global LR and RF models. While the prediction times of the LR models were generally faster than the RF models, the global approach was always the fastest, followed by the local and hybrid approaches.

**Table 1:** Average results from the leave-one-patient-out cross-validations for each model.

Model	Locality	mean ROC AUC	mean Dice	mean Sensitivity	mean Specificity	mean Prediction Time (s)
LR	Global	0.809±0.110**	0.322±0.218**	0.386±0.243**	0.959±0.047	**0.055±0.031**
LR	Local	0.861±0.109**	0.337±0.221**	**0.448±0.254**	0.955±0.041**	0.062±0.015*
LR	Hybrid	**0.872±0.092**	0.348±0.221	0.444±0.252	0.955±0.047**	0.126±0.038**
RF	Global	0.789±0.104**	0.319±0.215**	0.361±0.218**	**0.965±0.037**	29.762±6.857**
RF	Local	0.845±0.099**	0.311±0.208**	0.404±0.208**	0.956±0.030**	704.859±146.593**
RF	Hybrid	0.859±0.089**	**0.353±0.220**	0.415±0.231**	0.964±0.034	736.284±148.309**

Average ROC AUC values, Dice coefficients, sensitivity, specificity, and prediction time values for one dataset from the leave-one-patient-out cross-validation for each model. Best results according to each metric are highlighted in bold. Significant differences to this best-performing method computed with a one-sided paired student’s t-test are marked with a star (*) for a confidence interval of 95% (p < 0.05) and two stars (**) for a confidence interval of 99% (p < 0.01). Nominal p-values are reported without correction for multiplicity, similarly as in [23].

**Fig 1:**
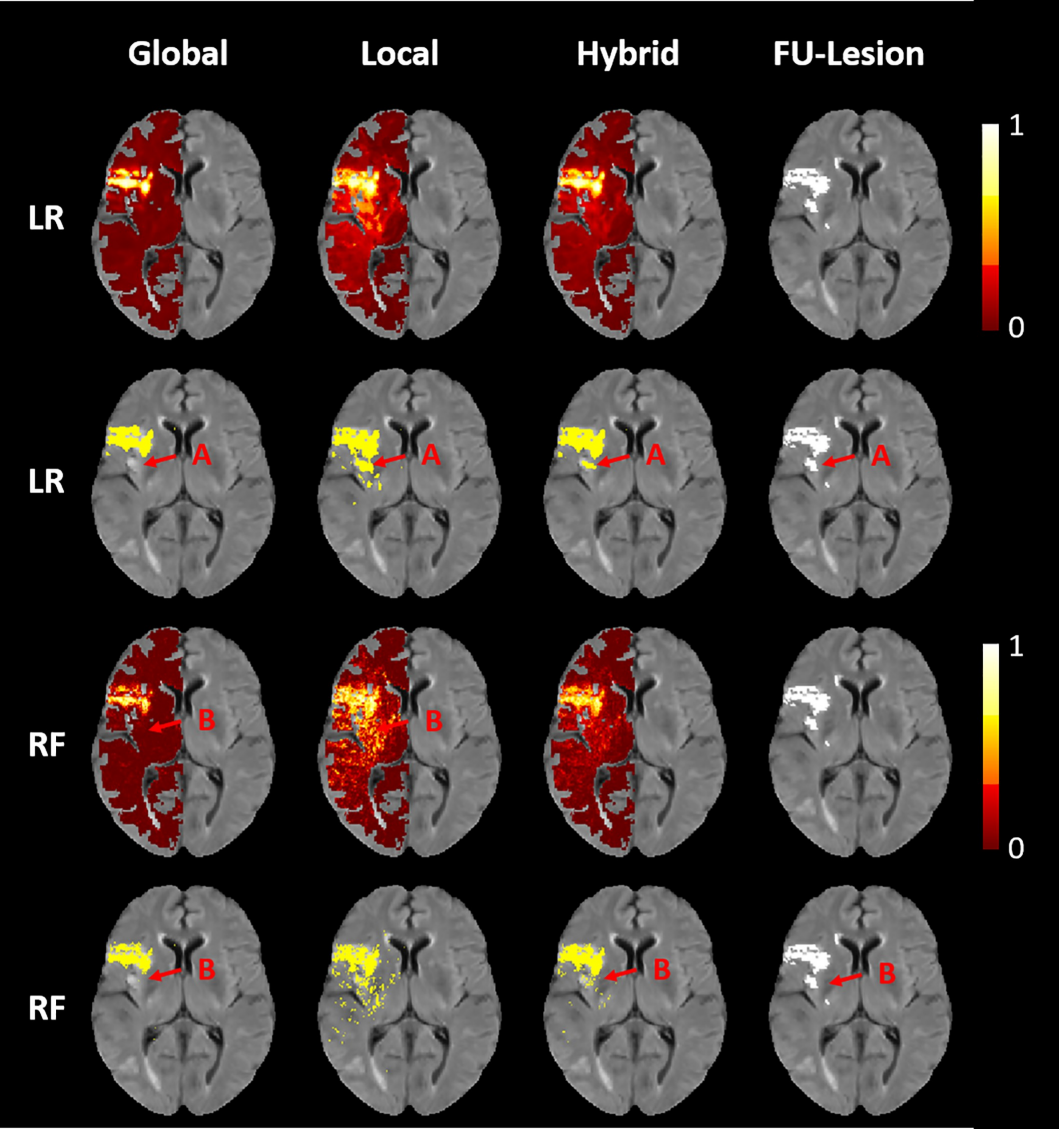
Final infarct outcome predictions for a selected patient. Images of the final infarct outcome predictions (first and third row) and binarized masks (second and fourth row) for the global, local, and hybrid (left to right) LR and RF (top to bottom) models for a selected patient and the corresponding true follow-up lesion outcome shown on the far right. In areas where the global models underestimates the lesion, the local models show higher infarct probabilities leading to better fits of the binary prediction masks, with the true follow-up lesion outcome; for both, LR (A) and RF (B). In addition, the global LR and RF models show a smooth coherent infarct prediction, whereas the local approach, especially the RF model, is slightly more scattered. Nevertheless, the dispersion of the local approach is concentrated on the actual infarct regions, so that the hybrid prediction is not only smoother than the local approaches, but also leads to the overall best qualitative results.

An illustration of the final lesion predictions of the global, local, and hybrid LR an RF models for a selected patient can be found in Fig 1. Here, it can be seen that the global approach results in predictions with the strongest contrast of all approaches differentiating lesion from non-lesion tissue while the local approach generally tends to produce noisier but more sensitive outcome prediction masks. Therefore, combining the global approach with higher specificity with the local approach with higher sensitivity in the hybrid approach leads to overall better results.

In addition, some of the coefficients of the local logistic regression models were not normally distributed, so that median values of the coefficients are provided in S1 Table. Overall, it can be seen that the coefficients of the local logistic regression models differ considerably between brain regions, which supports the benefit of using local tissue outcome prediction models.

## Discussion

The main finding from this study is that it is beneficial to use locally trained machine learning models for lesion outcome prediction. We compared global LR and RF models with the proposed local tissue outcome prediction approach, which combines many local tissue outcome prediction models (augmented with the global model) and with the hybrid approach, which is simply the average of the models resulting from the global and local approaches. For both algorithms (LR and RF), the global approach generally performed worse than the local approach, which in turn was outperformed by the hybrid approach. The global RF model yielded a slightly higher Dice coefficient than the local RF model. Only in case of the sensitivity, the global approach revealed consistent (but small) performance gains compared to the local and hybrid approaches.

### Combining local and global approaches

One of the main limitations of the proposed local approach is that despite the comparably large database available for this work, it was not possible to train local models for approximately 28% of all brain tissue voxels in MNI space. Thus, it is still an open question how good a pure local approach without any enhanced global predictions would perform. Potential ways to increase the number of local models include modifying the enrichment process, *e*.*g*. the radius used, or train the local models with more datasets of acute ischemic stroke patients. However, when using a global model to substitute local predictions, it is beneficial to use a global model that was trained using similar conditions compared to the local models and not using artificially balanced training datasets. Balanced global training datasets might artificially produce higher lesion likelihoods in contrast to predictions from models, which were trained on imbalanced data (like the local models) so that the two prediction types cannot be combined easily without normalization. Therefore, when down sampling the training data for the global approach, we stratified the data based on the follow-up outcome. Although the local training data of the local models was enriched with additional voxels with the aim to balance lesion and non-lesion voxels, the individual training datasets showed a varying ratio of lesion and non-lesion voxels due to the scarcity of lesion voxels and the spatial lesion distribution.

### Local training data enrichment

Similar to the selection of hyperparameters for machine learning models, the method used for training data enrichment is of particular importance for the training and subsequent accuracy of the local tissue outcome prediction models. Within this context, the number of observations, the local class balance at different positions, and the "locality" of the voxels play an important role. For example, it is important that the ratio of lesion and non-lesion training voxels for the different local models are as similar as possible. Otherwise, two models might not only differ because of the spatial conditions at the voxel positions, but also because of the higher ratio of lesion-voxels in the training data in one of the models. The latter usually leads to higher average predictions [24]. Therefore, if differences between the training datasets of the individual models are too large, they are more difficult to compare (without introducing an additional normalization step) and–as we are optimizing the performance for the joint prediction of a patient's tissue outcome–the determination of a common threshold for binarization becomes increasingly difficult. In this study, we have used a small radius to ensure that the training data is truly local and reduce variation in outcome prevalence by accumulating one non-lesion and one lesion voxel (where available). Furthermore, we have used a minimum group size of three non-lesion voxels to ensure that each local model is trainable and that enough models can be trained at the same time. Nevertheless, it remains an open question what minimum group size represents the best trade-off between the number of trainable local models and accuracy. It would also be possible to use a dynamic radius, possibly in combination with up-sampling, to achieve a class balance in the training data, as it was previously found that balanced training data leads to better prediction results in case of global tissue outcome prediction models [25].

### Scalability of the local approach

As the training procedures of the local models are completely independent from each other, they can be run in parallel, which allows to apply this approach using the full voxel information from almost any given number of patient datasets. The number of observations used per local model generation is equal to the number of included patients, which is magnitudes smaller than a global training set composed of the complete voxel information available. Therefore, no sub-sampling as used in case of the global random forest model is required. Practically, this also allows to use any advanced machine learning model for tissue outcome prediction, *i*.*e*. not just logistic regression or random forest, which were primarily chosen here, since they are computationally fast to train compared to other machine learning models.

### MNI registration

One of the main imitations of the local tissue outcome modeling approach is the required registration to the MNI space, which also adds some computational costs to the prediction. Nevertheless, all cases that successfully passed the other preprocessing steps could also be successfully registered to the MNI space using ANTs in this study. Although the voxel resolution is increased by a factor of approximately 20 by registering the patient scans into the MNI space, the actual lesion outcome prediction is not considerably slower compared to the global approach in case of the LR model. This is because the local LR model is essentially a coefficient matrix so that the local prediction can be obtained by simple matrix multiplication and application of the LR formula. In case of the RF approach, the global prediction is already comparatively slow with 24.2 seconds but still sufficiently fast. The local approach is considerably slower with a time of 705 seconds achieved with the current implementation. An advantage of the local approach, however, is not only the possibility of parallelization of the training but also of the prediction task so that the prediction time specified for one CPU core can be minimized trivially by using additional cores.

### Implementation of spatial features into currently used models

The results suggest that the positional information improves tissue outcome predictions. Therefore, integration of the position should be considered in future tissue outcome models as well. However, it does not necessarily have to be implemented the same way as described in this work. One option would be to build regional machine learning models for each brain region of a brain parcellation (*e*.*g*. Harvard-Oxford brain atlas). This would reduce the number of local models considerably while still allowing to make local predictions. Within this context, it should also be mentioned that gray and white matter differences are only implicitly modelled by the proposed approach. Due to the enrichment method using the search within a small radius, there is a chance that white and gray matter information might be mixed in the local training sets. Thus, one potential improvement would be to specifically search for voxels from the same tissue or the same region.

For an inclusion of spatial factors in previously described models, information about atlas regions could also be integrated as ordinary features within global approaches. For example, direct information on the location, such as the position, brain region, and tissue type (white or grey) of a respective voxel can be coded as additional features. In addition, the relative position to relevant positions, such as the distance of a voxel to the ischemic core, could be used to improve previously described global tissue outcome models.

## Conclusion

The findings from this study suggest that it is beneficial to employ locally trained machine learning models for lesion outcome prediction. The proposed method paves the way to use more sophisticated machine learning models for tissue outcome prediction that are too slow when trained using globally extracted voxel-wise training data.

## Supporting information

S1 Table Different median coefficient values per volume of interest for the local logistic regression approach. (DOCX)

## References

[1] Feigin VL, Krishnamurthi R V, Parmar P, et al Update on the global burden of ischemic and hemorrhagic stroke in 1990–2013: The GBD 2013 study. *Neuroepidemiology* 2015; 45: 161–176. 10.1159/000441085 26505981 PMC4633282

[2] Forkert ND, Kaesemann P, Treszl A, et al Comparison of 10 TTP and Tmax estimation techniques for MR perfusion-diffusion mismatch quantification in acute stroke. *AJNR Am J Neuroradiol* 2013; 34: 1697–1703. 10.3174/ajnr.A3460 23538410 PMC7965638

[3] Sussman ES, Connolly ES. Hemorrhagic transformation: A review of the rate of hemorrhage in the major clinical trials of acute ischemic stroke. *Front Neurol* 2013; 4 6: 1–8. 10.3389/fneur.2013.00001 23772220 PMC3677128

[4] Winder AJ, Siemonsen S, Flottmann F, et al Technical considerations of multi-parametric tissue outcome prediction methods in acute ischemic stroke patients. *Sci Rep* 2019; 9: 1–12. 10.1038/s41598-018-37186-2 31519923 PMC6744509

[5] Wang D, Wang Y. Tissue window, not the time window, will guide acute stroke treatment. *Stroke Vasc Neurol* 2019; 4: 1–2. 10.1136/svn-2018-000211 31105971 PMC6475083

[6] Turc G, Bhogal P, Fischer U, et al European Stroke Organisation (ESO)—European Society for Minimally Invasive Neurological Therapy (ESMINT) Guidelines on Mechanical Thrombectomy in Acute Ischemic Stroke. *Journal of NeuroInterventional Surgery*. Epub ahead of print 2019. 10.1136/neurintsurg-2018-014569 31152058

[7] Chen F. Magnetic resonance diffusion-perfusion mismatch in acute ischemic stroke: An update. *World J Radiol* 2012; 4: 63 10.4329/wjr.v4.i3.63 22468186 PMC3314930

[8] Kidwell CS, Wintermark M, De Silva DA, et al Multiparametric MRI and CT models of infarct core and favorable penumbral imaging patterns in acute ischemic stroke. *Stroke* 2012; 44: 73–79. 10.1161/STROKEAHA.112.670034 23233383 PMC3558033

[9] d’Esterre CD, Boesen ME, Ahn SH, et al Time-dependent computed tomographic perfusion thresholds for patients with acute ischemic stroke. *Stroke* 2015; 46: 3390–3397. 10.1161/STROKEAHA.115.009250 26514186

[10] Sah RG, d’Esterre CD, Hill MD, et al Diffusion-Weighted MRI Stroke Volume Following Recanalization Treatment is Threshold-Dependent. *Clin Neuroradiol* 2019; 29: 135–141. 10.1007/s00062-017-0634-4 29051996

[11] Winzeck S, Hakim A, McKinley R, et al ISLES 2016 and 2017-Benchmarking Ischemic Stroke Lesion Outcome Prediction Based on Multispectral MRI. *Front Neurol* 2018; 9: 679 10.3389/fneur.2018.00679 30271370 PMC6146088

[12] Wu O, Koroshetz WJ, Østergaard L, et al Predicting tissue outcome in acute human cerebral ischemia using combined diffusion- and perfusion-weighted MR imaging. *Stroke* 2001; 32: 933–942. 10.1161/01.str.32.4.933 11283394

[13] Nielsen A, Hansen MB, Tietze A, et al Prediction of Tissue Outcome and Assessment of Treatment Effect in Acute Ischemic Stroke Using Deep Learning. *Stroke* 2018; 49: 1394–1401. 10.1161/STROKEAHA.117.019740 29720437

[14] Arakawa S, Wright PM, Koga M, et al Ischemic Thresholds for Gray and White matter: A Diffusion and Perfusion Magnetic Resonance Study. *Stroke* 2006; 37: 1211–1216. 10.1161/01.STR.0000217258.63925.6b 16574931

[15] Siemonsen S, Forkert ND, Hansen A, et al Spatial distribution of perfusion abnormality in acute MCA occlusion is associated with likelihood of later recanalization. *J Cereb Blood Flow Metab* 2014; 34: 813–819. 10.1038/jcbfm.2014.13 24473482 PMC4013754

[16] Forkert ND, Cheng B, Kemmling A, et al ANTONIA perfusion and stroke: A software tool for the multi-purpose analysis of MR perfusion-weighted datasets and quantitative ischemic stroke assessment. *Methods Inf Med* 2014; 53: 469–481. 25301390 10.3414/ME14-01-0007

[17] Avants BB, Tustison NJ, Song G, et al A Reproducible Evaluation of ANTs Similarity Metric Performance in Brain Image Registration. *Neuroimage* 2011; 54: 2033–2044. 10.1016/j.neuroimage.2010.09.025 20851191 PMC3065962

[18] Kemmling A, Flottmann F, Forkert ND, et al Multivariate dynamic prediction of ischemic infarction and tissue salvage as a function of time and degree of recanalization. *J Cereb Blood Flow Metab* 2015; 35: 1397–1405. 10.1038/jcbfm.2015.144 26154867 PMC4640330

[19] Flottmann F, Broocks G, Faizy TD, et al CT-perfusion stroke imaging: A threshold free probabilistic approach to predict infarct volume compared to traditional ischemic thresholds. *Sci Rep* 2017; 7: 1–10. 10.1038/s41598-016-0028-x 28751692 PMC5532266

[20] R Core Team. R: A Language and Environment for Statistical Computing, https://www.r-project.org/ (2017).

[21] Obuchowski NA. Radiology Characteristic Curves and Their Use in Radiology. *Radiology* 2003; 229: 3–8. 10.1148/radiol.2291010898 14519861

[22] Zou KH, Warfield SK, Bharatha A, et al Statistical Validation of Image Segmentation Quality Based on a Spatial Overlap Index. *Acad Radiol* 2004; 11: 178–89. 10.1016/s1076-6332(03)00671-8 14974593 PMC1415224

[23] Maier O, Schröder C, Forkert ND, et al Classifiers for Ischemic Stroke Lesion Segmentation: A Comparison Study. *PLoS One* 2015; 10: 1–16. 10.1371/journal.pone.0145118 26672989 PMC4687679

[24] Hosmer DW, Lemeshow S, Sturdivant RX. *Applied Logistic Regression: Third Edition*. 2013 Epub ahead of print 2013. 10.1002/9781118548387

[25] Jonsdottir KY, Østergaard L, Mouridsen K. Predicting Tissue Outcome From Acute Stroke Magnetic Resonance Imaging: Improving Model Performance by Optimal Sampling of Training Data. *Stroke* 2009; 40: 3006–3011. 10.1161/STROKEAHA.109.552216 19608995

