# Supplementary material for: Localized prediction of tissue outcome in acute ischemic stroke patients using diffusion- and perfusion-weighted MRI datasets
Source: PLoS One. 2020 Nov 5;15(11):e0241917. doi: 10.1371/journal.pone.0241917 (PMC7643995; doi:10.1371/journal.pone.0241917)
Supplement: S1 Table — (DOCX) [file pone.0241917.s001.docx]

**S1 Table.** **Different median coefficient values per volume of interest for the local logistic regression approach.**

| VOI | Intercept | ADC | CBF | CBV | MTT | Tmax |
| --- | --- | --- | --- | --- | --- | --- |
| Caudate | 0.8011  ±1.4799 | -0.0017  ±0.0013 | -1.8685  ±1.3897 | 0.5217  ±0.6201 | 0.0415  ±0.0723 | 0.0217  ±0.0389 |
| Cerebellum | -0.2827  ±1.6072 | -0.002  ±0.0016 | -0.2569  ±0.8302 | -0.0837  ±0.4689 | 0.1319  ±0.106 | 0.0129  ±0.0413 |
| Frontal Lobe | -0.87  ±1.3517 | -0.001  ±0.0014 | -0.5195  ±1.194 | -0.184  ±0.641 | 0.1818  ±0.1313 | 0.0583  ±0.0516 |
| Insula | -0.7254  ±1.3496 | -0.0009  ±0.001 | -0.3134  ±0.7195 | 0.0431  ±0.3216 | 0.0766  ±0.0506 | 0.0551  ±0.0276 |
| Occipital Lobe | 0.2258  ±1.606 | -0.0022  ±0.0016 | -0.6754  ±1.6441 | 0.0874  ±0.7144 | 0.1407  ±0.1163 | 0.0532  ±0.0449 |
| Parietal Lobe | -0.3865  ±1.2535 | -0.0012  ±0.0012 | -0.3958  ±1.1542 | -0.1272  ±0.705 | 0.1205  ±0.1022 | 0.0489  ±0.0354 |
| Putamen | 0.3894  ±1.3084 | -0.0018  ±0.0012 | -0.8067  ±0.8587 | 0.1076  ±0.3898 | 0.0775  ±0.074 | 0.044  ±0.0352 |
| Temporal Lobe | -0.4691  ±1.0631 | -0.0011  ±0.0011 | -0.4013  ±0.6622 | 0.002  ±0.3294 | 0.1315  ±0.078 | 0.0452  ±0.0287 |
| Thalamus | 1.195  ±1.283 | -0.0022  ±0.0014 | -0.528  ±2.0799 | -0.2935  ±0.8333 | 0.1895  ±0.114 | 0.0298  ±0.0482 |
| Global | -0.043  ±0.027 | -0.0031  ±0 | -0.1184  ±0.0117 | -0.2889  ±0.0211 | 0.2236  ±0.0021 | 0.0856  ±0.0001 |

Median coefficient values of the local logistic regression models trained at positions within the regarding volume of interest (VOI). Due to non-normal distribution, median values and the mean absolute deviation from the median are reported for the local model coefficients. For comparison, the unique point estimators of the coefficients and their standard deviations from the global logistic regression model are reported in the last row of the table.
